# Assessment of WHO/INRUD core drug use indicators in two tertiary care hospitals of Bahawalpur, Punjab, Pakistan

**DOI:** 10.1186/s40545-016-0076-4

**Published:** 2016-09-22

**Authors:** Muhammad Atif, Muhammad Rehan Sarwar, Muhammad Azeem, Danial Umer, Abdul Rauf, Arslan Rasool, Muhammad Ahsan, Shane Scahill

**Affiliations:** 1 Department of Pharmacy, The Islamia University of Bahawalpur, Bahawalpur, Pakistan; 2 School of Management, Massey University, Auckland, New Zealand

**Keywords:** Rational, Use of drugs, Prescribing pattern, Patient-care, Facility-specific

## Abstract

**Background:**

Medicines are a main therapeutic intervention provided within hospitals and their proper use in the outpatient setting is important for patients and the community. The objective of this study was to evaluate drug use patterns in the outpatient departments (OPDs) of two tertiary care hospitals (Bahawal Victoria Hospital and Civil Hospital) in the Bahawalpur district of the Punjab province of Pakistan by employing the standard World Health Organization/International Network of Rational Use of Drugs (WHO/INRUD) drug use indicators.

**Methods:**

A descriptive, cross-sectional study design was employed. For assessing the prescribing indicators a sample of 2400 prescriptions were systematically reviewed out of a total of 1,560,000 prescriptions written from 1st April 2014 to 31st March 2015. A total of 600 randomly selected patients and all pharmacy personnel were observed and interviewed to investigate the patient-care and facility-specific indicators. We used the published ideal standards for each of the WHO/INRUD indicators for comparison purposes.

**Results:**

Among the prescribing indicators, the average number of drugs per prescription was 2.8 (SD = 1.3), the drugs prescribed by generic name were 56.6 %, the encounters with an antibiotic prescribed were 51.5 %, no injections were prescribed and 98.8 % of the drugs prescribed were from the Essential Drugs List (EDL). Among the patient-care indicators, the average consultation time was 1.2 min (SD = 0.8), the average dispensing time was 8.7 s (SD = 4.9), the percentage of drugs actually dispensed was 97.3 %, the percentage of drugs adequately labeled was 100 % and the patients’ knowledge of correct dosage schedule was 61.6 %. Among the facility-specific indicators, all OPDs had a copy of the EDL and 72.4 % of the key drugs were available in stock.

**Conclusion:**

Irrational use of drugs was observed in both OPDs. Polypharmacy, brand prescribing, over-prescribing of antibiotics, short consultation and dispensing times, lack of patients’ knowledge about prescribed medicines and unavailability of all key drugs in stock were the major issues that need attention of the healthcare authorities. This study necessitates the requirement to implement the relevant WHO recommended core interventions to promote rational use of medicines in these hospital-based OPDs.

## Background

The World Health Organization (WHO) defines rational use of medicines as “patients receive medications appropriate to their clinical needs, in doses that meet their own individual requirements, for an adequate period of time, and at the lowest cost to them and their community” [1]. According to the WHO, more than 50 % of all medicines are prescribed, dispensed or sold inappropriately [2]. As a result, patients are unable to follow their treatment plan correctly. One of the consequences of irrational use of medicines is lack of patients’ confidence in the healthcare system. In the developing countries, for example Pakistan, the problem is exacerbated by limited resources and inadequate drug policy [3].

The most common causes of inappropriate use of medicines are; self-medication, polypharmacy, inappropriate use of antibiotics, overuse of injectable medicines and prescribing of medicines outside of usual clinical practice guidelines [2]. Some other causes of irrational use of medicines include knowledge, attitudes and practices of patients and practitioners, the working environment, the drug supply system, legal regulations, information and misinformation about the medicines and profit intentions of the pharmaceutical companies [4–8]. Unsafe and ineffective treatment, prolongation of disease state, harm and distress to the patients, increased cost of treatment, aggravation of chronic conditions for example diabetes, hypertension, epilepsy and neurological disorders, and antibiotic resistance are some of the consequences of the irrational use of medicines [9].

The fundamental steps to limit the irrational use of medicines is to identify the types, extent and reasons of their irrational use [2]. In the 1990′s, WHO in collaboration with the International Network of Rational Use of Drugs (INRUD) developed standard indicators to evaluate drug use practices at healthcare centers [10]. Though originally developed to assess drug use patterns in primary healthcare centers, these indicators have been used at tertiary healthcare facilities for similar purposes [11–13]. The indicators called “core drug use indicators” are classified into prescribing, patient-care and facility-specific indicators (Table 1).

**Table 1:** Core drug use indicators and their optimal values

Core drug use indicators	Optimal values
Prescribing Indicators	
Average number of drugs prescribed per patient encounter	1.6–1.8
Percent medicines prescribed by generic name	100
Percent encounters with an antibiotic prescribed	20.0–26.8
Percent encounters with an injection prescribed	13.4–24.1
Percent medicines prescribed from essential medicines list or formulary	100
Patient-Care Indicators	
Average consultation time (minutes)	≥10
Average dispensing time (seconds)	≥90
Percent medicines actually dispensed	100
Percent medicines adequately labeled	100
Percent patients with knowledge of correct doses	100
Facility-Specific Indicators	
Availability of essential medicines list or formulary to practitioners	100
Percent key medicines available	100

Note: Core drug use indicators are obtained from World Health Organization source [2, 10]

The purpose of this study was to investigate the drug use patterns at ten outpatient departments (OPDs) in each of the two tertiary care hospitals in the Bahawalpur district of Southern Punjab, Pakistan. The findings are able to be used as a benchmark for the healthcare facilities and as a basis for further follow-up of quality of drug use. In the context of Pakistan, the findings of this study will further help policymakers to take necessary measures to stimulate appropriate use of medicines.

## Methods

### Study setting

The study was conducted in the Bahawal Victoria Hospital (BVH) and the Civil Hospital of Bahawalpur district of the Punjab province of Pakistan. Ten OPDs from each of the two hospitals were included. The BVH, located in the central part of the city, is a 1600-bed tertiary care hospital with all standard medical and surgical facilities. The Civil Hospital is newly established 400-bed tertiary care hospital.

### Study design and outcome measures

This descriptive, cross-sectional study was conducted to evaluate the performance of the selected OPDs in three general areas related to rational drug use; prescribing, patient-care and facility. The optimal values for the prescribing [11], patient-care and facility-specific indicators [9, 14] were adopted from previous studies. For this study, the optimal values for the consultation and dispensing times were set as ≥10 min and ≥90 s, respectively (Table 1).

Zhang and Zhi developed an index system to gauge the performance of a healthcare system in terms of drug utilization [14, 15]. For the calculation of non-polypharmacy, rational antibiotic and injection safety indices, the following formula was used;

$$ Index=\frac{\mathrm{Optimal}\ \mathrm{value}}{\mathrm{Observed}\ \mathrm{value}} $$

All other indices (index of generic name, index of Essential Drugs List (EDL), consultation time index, dispensing time index, index of drugs actually dispensed, index of labelling of drugs, index of patients’ knowledge, index of EDL availability and index of key drugs availability in stock) were calculated by the following formula;

$$ Index=\frac{\mathrm{Observed}\kern0.5em \mathrm{value}}{\mathrm{Optimal}\ \mathrm{value}} $$

The optimal index for all indicators was set as 1. The values closer to 1 indicated rational drug use and vice versa. The Index of Rational Drug Prescribing (IRDP) was calculated for all OPDs by adding the index values of all prescribing indicators. Based on the IRDP values, the OPDs were ranked from 1 to 10 (rank 1 for the higher IRDP value and rank 10 for the lower IRDP). In a similar fashion, the Index of Rational Patient-Care Drug Use (IRPCDU) and the Index of Rational Facility-Specific Drug Use (IRFSDU) were calculated. Finally, the Index of Rational Drug Use (IRDU) was calculated for all OPDs by adding up the total of IRDP, IRPCDU and IRFSDU. Subsequently, OPDs were ranked based on the IRDU indices. The OPD with higher IRDU value was considered the best performing OPD in terms of rational drug use, and was given the first rank.

### Data collection

The standard core drug use indicator forms were used to collect the data [10]. The WHO guidelines and methods were observed to ensure data reliability [10]. The data was collected during the period 1st December 2014 to 31st March 2015.

#### Prescribing indicators

Trained data collectors retrospectively selected 2,400 prescriptions (120 prescriptions per OPD) out of the total 1,560,000 prescriptions written during the 1 year period (i.e., 1st April 2014 to 31st March 2015). A systematic random sampling technique was used to collect the data and the sampling unit was the patient encounters at each of the OPDs [11]. To minimize the sampling bias (seasonal alterations or supply cycle of medicines), the encounters per year were uniformly divided into four quarters and 30 prescriptions were randomly selected from each quarter, irrespective of acute or chronic illnesses, including a mixture of health conditions and a range of patient ages.

#### Patient-care indicators

Patients visiting the OPDs, between 1st December 2014 and 31st March 2015 for diagnosis and treatment of health problems were invited to participate in the study. A total of 600 patients (30 patients per OPD) were randomly selected during the OPD hours. Trained data collectors explained the purpose of the study to the respondents and obtained their consent before data collection began. The consented patients were observed and interviewed to obtain the required information [10].

#### Facility-specific indicators

For the facility-specific indicators, all available pharmacy personnel were invited to participate in the study and the consented participants were interviewed to obtain the required information [10].

### Data analysis

Statistical Package for Social Sciences (IBM, SPSS Statistics for Windows, version 21.0. Armonk, NY: IBM Corp.) was used for data analysis. Descriptive statistics such as frequencies, percentages, average/mean, and standard deviation were used to present the data.

## Results

### Prescribing indicators

The average number of drugs per encounter was 2.82 (SD = 1.3) and percentage of drugs prescribed by the generic name was 56.6 % (SD = 1.2). Over half (51.5 %, SD = 0.5) of the prescriptions had an antibiotic prescribed. There was no injection encounter recorded. The percentage of drugs prescribed from the EDL was 98.8 % (SD = 1.3) (Table 2).

**Table 2:** WHO/INRUD prescribing indicators in two tertiary care hospitals of Bahawalpur (*N* = 2400)

OPD wards	Prescribing indicators									
	Average drugs/encounter		% drugs prescribed by generic name		% encounters with an antibiotic prescribed		% encounters with an injection prescribed		% drugs prescribed from EDL/formulary	
	BVH	Civil	BVH	Civil	BVH	Civil	BVH	Civil	BVH	Civil
Gynecology	3.1	2.8	66.0	51.3	38.0	43.3	0.0	0.0	100	87.1
Ophthalmology	1.2	1.6	28.2	84.1	48.0	71.6	0.0	0.0	100	99.4
Dermatology	2.2	2.8	44.2	54.4	48.0	35.0	0.0	0.0	100	98.8
ENT	3.2	2.8	72.5	62.2	80.0	55.8	0.0	0.0	100	100
Surgery	3.2	2.7	62.5	55.7	86.0	28.3	0.0	0.0	100	99.3
Pulmonology	3.7	2.0	55.6	56.0	54.1	6.6	0.0	0.0	100	98.3
Dental	2.8	2.5	45.1	39.3	95.0	95.0	0.0	0.0	100	99.7
Cardiology	4.6	4.1	52.8	67.0	0.0	10.0	0.0	0.0	100	95.5
Pediatrics	2.5	3.0	57.4	53.5	65.0	84.1	0.0	0.0	100	98.9
Medical	3.2	2.5	65.1	58.6	39.0	47.5	0.0	0.0	100	100
Mean	3.0	2.7	54.9	58.2	55.3	47.7	0.0	0.0	100	97.7
MEAN (SD)^a^	2.8 (1.3)		56.6 (1.2)		51.5 (0.5)		0.0 (0.0)		98.8 (1.3)	

^a^Calculated based on the total sample size, *OPD* outpatient department, *EDL* essential drugs list, *BVH* Bahawal Victoria Hospital, *ENT*, ear, nose & throat

### Patient-care and facility-specific indicators

The average consultation time was 1.2 (SD = 0.8) minutes and the average dispensing time was 8.7 (SD = 4.9) seconds. 97.3 % (SD = 1.3) of drugs on the prescriptions were actually dispensed. All dispensed drugs were adequately labelled. Over two-thirds (61.6 %, SD = 0.6) of patients had the correct knowledge about the dosage of the medications they had been prescribed (knowledge about when and in what quantity the medicine should be taken). A copy of the EDL was available 100 % (SD = 0.0) of the time. Nearly three-quarters (72.4 %, SD = 0.4) of key drugs were available in stock (Table 3).

**Table 3:** WHO/INRUD patient-care and facility-specific indicators in two tertiary care hospitals of Bahawalpur (*N* = 600)

OPD wards	Patient-care and facility-specific indicators													
	Average consultation time (minutes)		Average dispensing time (seconds)		% drugs dispensed		% drugs adequately labeled		% patients knowledgeable		% copy of EDL		% key drugs in the stock	
	BVH	Civil	BVH	Civil	BVH	Civil	BVH	Civil	BVH	Civil	BVH	Civil	BVH	Civil
Gynecology	2.2	0.9	7.8	6.7	100	100	100	100	47.0	73.0	100	100	100	80
Ophthalmology	0.7	0.6	4.8	5.9	100	100	100	100	87.0	67.0	100	100	43.0	86
Dermatology	1.1	0.7	9.0	5.1	67.6	100	100	100	73.0	70.0	100	100	50.0	75
ENT	0.9	0.7	7.5	6.3	94.9	100	100	100	27.0	50.0	100	100	100	71
Surgery	1.1	2.1	17.2	6.8	100	100	100	100	100	67.0	100	100	50.0	75
Pulmonology	1.3	2.1	7.6	8.0	96.7	100	100	100	70.0	47.0	100	100	71.0	71
Dental	1.7	0.7	8.0	6.2	100	100	100	100	40.0	60.0	100	100	75.0	50
Cardiology	1.6	2.4	18.6	8.6	100	100	100	100	67.0	50.0	100	100	75.0	75
Pediatrics	1.4	0.7	6.1	7.4	86.0	100	100	100	47.0	60.0	100	100	100	67
Medical	1.1	0.7	18	7.4	100	100	100	100	67.0	63.0	100	100	75.0	58
Mean	1.3	1.1	10.5	6.8	94.5	100	100	100	62.5	60.7	100	100	73.9	70.8
MEAN (SD)^a^	1.2 (0.8)		8.7 (4.9)		97.3 (1.3)		100 (0.0)		61.6 (0.6)		100 (0.0)		72.4 (0.4)	

^a^Calculated based on the total sample size, *OPD* outpatient department, *EDL* essential drugs list, *BVH* Bahawal Victoria Hospital, *ENT* ear, nose & throat

The IRDP values show that the gynecology OPD was performing well in the BVH and the pulmonology OPD was the leading department in the Civil Hospital. Similarly, the IRPCDU values indicated that the surgery OPDs in both hospitals showed better results compared with all other OPDs. The gynecology, ENT and pediatrics OPDs in the BVH and the ophthalmology OPD in the Civil Hospital showed relatively better results with regard to facility-specific indicators. Overall, the IRDU values showed that the gynecology OPD in the BVH and the pulmonology OPD in the Civil Hospital were the best performing OPDs (Table 4) in terms of core drug indicators.

**Table 4:** Index of rational drug use in two tertiary care hospitals of Bahawalpur

IRDU		OPDs									
		Gynecology	Ophthalmology	Dermatology	ENT	Surgery	Pulmonology	Dental	Cardiology	Pediatrics	Medical
Prescribing Indicators											
Non-polypharmacy index	BVH	0.58	1.00	0.82	0.56	0.56	0.49	0.64	0.40	0.72	0.56
	Civil	0.64	1.00	0.64	0.64	0.67	0.90	0.72	0.44	0.60	0.72
Generic name index	BVH	0.66	0.28	0.44	0.72	0.62	0.56	0.45	0.53	0.57	0.65
	Civil	0.51	0.84	0.54	0.62	0.56	0.56	0.39	0.67	0.53	0.59
Rational antibiotic index	BVH	0.71	0.56	0.56	0.34	0.31	0.49	0.28	1.00	0.41	0.69
	Civil	0.62	0.37	0.76	0.48	0.95	1.00	0.28	1.00	0.32	0.56
Injection safety index	BVH	1.00	1.00	1.00	1.00	1.00	1.00	1.00	1.00	1.00	1.00
	Civil	1.00	1.00	1.00	1.00	1.00	1.00	1.00	1.00	1.00	1.00
Essential drugs list index	BVH	1.00	1.00	1.00	1.00	1.00	1.00	1.00	1.00	1.00	1.00
	Civil	0.87	0.99	0.99	1.00	0.99	0.98	0.99	0.95	0.99	1.00
IRDP	BVH	3.95	3.84	3.82	3.62	3.49	3.54	3.37	3.93	3.70	3.90
	Civil	3.64	4.20	3.93	3.74	4.17	4.44	3.38	4.06	3.44	3.87
Rank	BVH	1	4	5	7	9	8	10	2	6	3
	Civil	8	2	5	7	3	1	10	4	9	6
Patient-Care Indicators											
Consultation time index	BVH	0.22	0.07	0.11	0.09	0.11	0.13	0.17	0.16	0.14	0.11
	Civil	0.09	0.06	0.07	0.07	0.21	0.21	0.07	0.24	0.07	0.07
Dispensing time index	BVH	0.09	0.05	0.10	0.08	0.19	0.08	0.09	0.21	0.07	0.20
	Civil	0.07	0.06	0.06	0.07	0.08	0.09	0.07	0.10	0.08	0.08
Dispensed drugs index	BVH	1.00	1.00	0.67	0.94	1.00	0.97	1.00	1.00	0.86	1.00
	Civil	1.00	1.00	1.00	1.00	1.00	1.00	1.00	1.00	1.00	1.00
Labeled drugs index	BVH	1.00	1.00	1.00	1.00	1.00	1.00	1.00	1.00	1.00	1.00
	Civil	1.00	1.00	1.00	1.00	1.00	1.00	1.00	1.00	1.00	1.00
Patients’ knowledge index	BVH	0.47	0.87	0.73	0.27	1.00	0.70	0.40	0.67	0.47	0.67
	Civil	0.73	0.67	0.70	0.50	0.67	0.47	0.60	0.50	0.60	0.63
IRPCDU	BVH	2.78	2.99	2.61	2.38	3.30	2.88	2.66	3.04	2.54	2.98
	Civil	2.89	2.79	2.83	2.64	2.96	2.77	2.74	2.84	2.75	2.78
Rank	BVH	6	3	8	10	1	5	7	2	9	4
	Civil	2	5	4	10	1	7	9	3	8	6
Facility-Specific Indicators											
Index of EDL	BVH	1.00	1.00	1.00	1.00	1.00	1.00	1.00	1.00	1.00	1.00
	Civil	1.00	1.00	1.00	1.00	1.00	1.00	1.00	1.00	1.00	1.00
Index of key drugs in stock	BVH	1.00	0.43	0.50	1.00	0.50	0.71	0.75	0.75	1.00	0.75
	Civil	0.80	0.86	0.75	0.71	0.75	0.71	0.50	0.75	0.67	0.58
IRFSDU	BVH	2.00	1.43	1.50	2.00	1.5	1.71	1.75	1.75	2.00	1.75
	Civil	1.80	1.86	1.75	1.71	1.75	1.71	1.50	1.75	1.67	1.58
Rank	BVH	1	5	4	1	4	3	2	2	1	2
	Civil	2	1	3	4	3	4	7	3	5	6
Grand Total											
IRDU	BVH	8.73	8.26	7.93	8.00	8.29	8.13	7.78	8.72	8.24	8.63
	Civil	8.33	8.85	8.51	8.09	8.88	8.92	7.62	8.65	7.86	8.23
Rank	BVH	1	5	9	8	4	6	10	2	7	3
	Civil	6	3	5	8	2	1	10	4	9	7

*OPD* outpatient department, *EDL* essential drugs list, *BVH* Bahawal Victoria Hospital, *ENT*, ear, nose & throat, *IRDP* index of rational drug prescribing, *IRPCDU*, index of rational patient-care drug use, *IRFSDU* index of rational facility-specific drug use, *IRDU* index of rational drug use

## Discussion

This study set out to assess drug use practices through analysis of the core indicators of the WHO/INRUD across a range of OPDs in two tertiary care hospitals in Pakistan. Though irrational prescribing practices exist all over the world [9], the gravity of the problem is high in low and middle income countries and so Pakistan makes for a sensible context in which to explore this issue. Our study reports: a higher number of drugs prescribed per prescription, over-use of antibiotics, low rate of prescribing by generic name and short dispensing and consulting times. Key drugs were available less than three-quarters (72.4 %) of the time. Patient knowledge of correct doses was found to be lower than like studies in other developing countries. On the positive side, the EDL was always available in these OPDs and 98.8 % of drugs prescribed were from the list. The implications of these findings are discussed in terms of previous studies and future policy, practice and research.

### Prescribing indicators

Our findings suggest the average number of drugs per prescription was higher than the admissible range and there was considerable over-prescribing of antibiotics. We also found that just over half (56.6 %) of drugs were prescribed by the generic name whilst the aim is for 100 % prescribing by generic. These findings are alarming for a number of reasons. Polypharmacy can adversely influence treatment outcomes in a way that patients are at a higher risk of adverse events [16]. Similarly, unnecessarily prescribed medicines and those prescribed by brand names can put extra financial pressure on patients as well as healthcare budgets [2]. According to the WHO, it is the aim that generic prescribing takes precedent over originator brand prescribing because it helps with improved communication and clarity amongst healthcare providers. Moreover, generic medicines are less expensive than originator branded medicines [17]. The over-use of antibiotics is occurring internationally and leads to increase in adverse drug reactions and hospitalization, but also contributes significantly to an increase in antibiotic resistance and Pakistan appears no different [18].

In our study, we found that injectable medicines were not prescribed and the EDL was followed in 98.8 % of the prescriptions. It is likely that injectable medicines were not prescribed because in Pakistan they are more commonly reserved for emergency cases, and in the context of this study, such patients would be managed in Accident and Emergency (A&E) departments, and A&E departments were not included in our study.

We have summarized the findings of closely related studies, irrespective of country of origin, to facilitate comparisons (Table 5). With regard to prescribing indicators, our findings are closely related to earlier studies in this field. For example, studies from Afghanistan (3.9) [19], India (5.6) [20], Ghana (4.8) [21] and Nigeria (5.2) [22] showed higher numbers of drugs per prescription. Similarly, in studies from Andorra (6 %) [23], Palestine (5.5 %) [24], Lebanon (2.9 %) [25] and India (11.5 %) [3], generics were less commonly prescribed than was seen in this study. In contrast to this, studies from Cambodia (99.8 %) [26], Timor-Leste (92 %) [27] and Ethiopia (98.7 %) [11] showed higher levels of generic prescribing, which might be the consequence of more effective and better enforced drug policies in these countries; or simply a lack of very expensive originator branded drugs.

**Table 5:** Summary of core drug use indicator studies from developed and developing countries

Indicators	Study reference									
	Developed countries					Developing countries				
	[14, 35]	[38]	[39]	[40]	[41]	[9]	[42]	[26]	[22]	[43]
Average no. of drugs per encounter	2.4	0.7	2.9	2.6	2.2	2.5	2.2	2.4	3.0	2.2
% Generic	61.2	----	17.7	14.3	4.4	95.4	74	99.8	63.1	99
% Antibiotic	32.2	10.4	39.1	26.2	13.5	39.2	37	66	54.2	43
% Injection	2	0.0	9.1	8.3	1.6	9.9	11	2.4	38.0	18
% drugs EDL	99.2	----	----	19.8	----	95.4	78	99.7	75.6	98.8
Average consultation time (minutes)	7.3	22.5	2.8	----	----	7.1	5.8	4.4	6.1	3.7
Average dispensing time (seconds)	100	----	54.6	----	----	47.4	17	234	18.1	37
% Drugs dispensed	99.6	----	97.9	----	----	95.9	66	100	99.1	84.5
% Drugs labeled	10	----	66.9	----	----	0.0	63	0.0	55.9	86.2
% Patient knowledge	79.3	70	26.9	----	----	94	54	55	86.5	81.7
% EDL availability	90	----	-----	----	----	80	50	100	100	100
% Drugs in stock	59.2	-----	------	----	----	78.3	55	86.6	91.7	86.5

Saudi Arabia [14, 35]; Sweden [38]; Kuwait [39]; Bahrain [40]; United Arab Emirates [41]; Egypt [9]; Brazil [42]; Cambodia [26]; Swaziland [22]; Mozambique [43]

### Patient-care indicators

The results of the current study demonstrate that the average consultation and dispensing times were 1.2 min and 8.7 s, respectively (dispensing time = time when a patient reaches and leaves the pharmacy counter. Waiting time not included). This is much less than the proposed optimal norms. The shorter consultation and dispensing times reported in our study could be a result of the large number of patients attending the OPDs in these two tertiary care hospitals. Insufficient consultation time leads to inappropriate patient examination and history taking and poor physician–patient interactions [9]. On the other hand, shorter dispensing time is insufficient to provide complete drug related information to the patients. Several studies from various under-developed countries reported almost similar findings [22, 28, 29]. However, studies from Nigeria [30], Nepal [31] and Ethiopia [32] showed better results probably because of adequate patient-to-healthcare worker ratio. According to our findings, 61.6 % of the patients had knowledge of correct dosage schedule for all prescribed drugs. This is much lesser than the optimal value of 100 %. It was higher than that reported in Tanzania (37.9 %) [33] and Malawi (27 %) [34] but much lower than high achieving nations such as Nigeria (93 %) [30] and Egypt (94 %) [9]. Patient’s knowledge about correct dosage is highly significant to avoid over use and abuse of drugs, and could prevent adverse events that ultimately affect patients’ health and quality of life.

In contrast to dispensing time, consultation time, and patients’ knowledge of correct dosage, the percentage of drugs actually dispensed and the drug labelling practices were close to the optimal values. This may be due to the fact that the medical professionals were aware of the drugs available in the hospital and followed appropriate guidelines with regards EDL use.

### Facility-specific indicators

The study revealed that all OPDs had a copy of the EDL which is in line with the proposed norm. However, only 72.4 % of key drugs were available in on hand in these OPDs. Shortage of any essential drugs in the hospital is disadvantageous for patients in that doctors may not be able to prescribe the correct drugs or they are limited to prescribing out-of-stock medicines which may pose extra fiscal burden on the patients’ through “out of pocket” expense. Similar to our findings, studies from Saudi Arabia (59.2 %) [35], Nigeria (62 %) [36], Malawi (67 %) [34] and Ethiopia (65 %) [32] showed lower values for availability of key drugs in the stock. In contrast, Tanzania (100 %) [37], Swaziland (91.7 %) [22], Nigeria (90.9 %) [22] and Nepal (90 %) [31] appear to report better results.

### Implications

In addition to the implications for literature (outlined previously) there are also considerations for policy, practice and future research. Our findings will benefit policy makers who have an interest in process improvement and service delivery performance. The finding that only 72.4 % of key drugs were available throughout the study warrants consideration by policy-makers, who may need to put incentives or legislation in place to ensure better access to these essential drugs. There are also areas of practice which could be improved on particularly around the potential over-use of antibiotics and the prescribing of drugs by generic name. The two hospitals would benefit from having guidelines in place for the optimal and responsible use of antibiotics which could be monitored and feedback provided on a regular basis to prescribers. An awareness campaign and incentives could also be put in place to increase the prescribing of drugs by generic name. This intervention could be set up as a program evaluation and add to the current research agenda in this area. One approach could be to implement and evaluate WHO recommended core interventions on a pilot scale to devise policies to achieve improved performance and the long-term benefits associated with this.

## Conclusion

Polypharmacy, brand prescribing, over-prescribing of antibiotics, short consultation and dispensing times, lack of patients’ knowledge about prescribed medicines and unavailability of all key drugs in stock were the major issues that need attention of the healthcare authorities. WHO recommended core interventions could be implemented on pilot scale to devise policies to achieve long-term benefits.

### Limitations

As with any study there are limitations which need to be considered.. First, our findings could not be generalized for the whole of Pakistan and should not be extrapolated to the international environment. The findings do however add to a growing literature, particularly around medicines use and pharmaceutical health systems in developing countries. It is also important to consider that a uniform healthcare policy is implemented throughout Pakistan and medical graduates from various institutions are working in the Bahawalpur district. It is expected that the practices would be similar to other tertiary care hospitals in Pakistan. Second, different OPDs in each of the tertiary care hospitals demonstrate varying degrees of optimal drug use and systems to support these. To address this bias, the mean values were calculated for each of the OPD, separately. Third, very limited data on core drug use indicators is available in tertiary care settings and we compare our findings with international data based on primary healthcare settings.

## Abbreviations

EDL, essential drugs list; INRUD, International Network for the Rational Use of Drugs; IRDP, index of rational drug prescribing; IRDU, index of rational drug use; IRFSDU, index of rational facility-specific drug use; IRPCDU, index of rational patient-care drug use; SPSS, statistical package for social sciences; WHO, World Health Organization

## References

[1] World Health Organization. The Rational use of drugs: report of the conference of experts, Nairobi, 25–29 November 1985. 1987. http://apps.who.int/medicinedocs/en/d/Js17054e/. Accessed October 18 2015

[2] World Health Organization. Promoting rational use of medicines: core components - WHO policy perspectives on medicines. 2002. http://apps.who.int/medicinedocs/en/d/Jh3011e/. Accessed October 18 2015

[3] Kshirsagar M, Langade D, Patil S, Patki P (1998). Prescribing patterns among medical practitioners in Pune, India. Bull World Health Organ.

[4] World Health Organization. The use of essential drugs: sixth report of the WHO Expert Committee. 1995. http://apps.who.int/iris/handle/10665/37340. Accessed October 18 2015 7597816

[5] Geest S, Hardon A, Whyte S (1991). Planning for essential drugs: are we missing the cultural dimension. Health Policy Plan.

[6] Hogerzeil HV (1995). Promoting rational prescribing: an international perspective. Br J Clin Pharmacol.

[7] Sachs L, Tomson G (1992). Medicines and culture--a double perspective on drug utilization in a developing country. Soc Sci Med.

[8] Abdo-Rabbo A, Haaijer-Ruskamp F, Basharahil K (2000). Baseline prescribing and health facility indicators in Yemen. J Fac Med Baghdad.

[9] Akl OA, El Mahalli AA, Elkahky AA, Salem AM (2014). WHO/INRUD drug use indicators at primary healthcare centers in Alexandria, Egypt. J Taibah Univers Med Sci.

[10] World Health Organization. How to investigate drug use in health facilities: selected drug use indicators. 1993. http://apps.who.int/medicinedocs/en/d/Js2289e/. Accessed October 18 2015

[11] Desalegn AA (2013). Assessment of drug use pattern using WHO prescribing indicators at Hawassa University teaching and referral hospital, south Ethiopia: a cross-sectional study. BMC Health Serv Res.

[12] Malangu N (1992). Indicators in a Zaire hospital. INRUD News.

[13] Isah A, Ohaju‐Obodo J, Isah E, Ozemoya O (1997). Drug use profile in a Nigerian city hospital. Pharmacoepidemiol Drug Saf.

[14] El Mahalli A (2012). WHO/INRUD drug prescribing indicators at primary health care centres in Eastern province, Saudi Arabia. East Mediterr Health J.

[15] Zhang Y, Zhi M (1995). Index system, appraising method for comprehensive appraisal. J North Jiaotong Univer.

[16] Hohl CM, Dankoff J, Colacone A, Afilalo M (2001). Polypharmacy, adverse drug-related events, and potential adverse drug interactions in elderly patients presenting to an emergency department. Ann Emerg Med.

[17] Atif M, Azeem M, Sarwar MR (2016). Potential problems and recommendations regarding substitution of generic antiepileptic drugs: a systematic review of literature. SpringerPlus.

[18] World Health Organization. Antimicorbial resistance. 2015. http://www.who.int/mediacentre/factsheets/fs194/en/. Accessed June 19 2016

[19] Ahmad F, Danish AS, Rahmani MR. Prescribing practice at private clinics in jalalabad, Afghanistan. INRUD News. 1995;5(2):21

[20] Akhtar M, Vohora D, Pillai K, Dubey K, Roy M, Najmi A (2012). Drug prescribing practices in paediatric department of a north indian university teaching hospital. Asian J Pharm Clin Res.

[21] Bosu W, Ofori-Adjei D (1999). An audit of prescribing practices in health care facilities of the Wassa West district of Ghana. West Afr J Med.

[22] S.O.N. Uzoma AL, Eva Ombaka. Drug Use studies in Church Facilities in Africa. INRUD News. 1995;5(1):20.

[23] Vallano A, Montane E, Arnau JM, Vidal X, Pallares C, Coll M (2004). Medical speciality and pattern of medicines prescription. Eur J Clin Pharmacol.

[24] Fattouh R, Abu HB (2010). Impact of using essential drug list: analysis of drug use indicators in Gaza Strip. East Mediterr Health J.

[25] Hamadeh GN, Dickerson LM, Saab BR, Major SC (2001). Common prescriptions in ambulatory care in Lebanon. Ann Pharmacother.

[26] Chareonkul C, Khun VL, Boonshuyar C (2002). Rational drug use in Cambodia: study of three pilot health centers in Kampong Thom Province. Southeast Asian J Trop Med Public Health.

[27] Stanley Chindove AX (2012). Nelson Martins. Medicines prescribing patterns for the treatment of common diseases at community health centres in 3 districts of Timor-leste. Int J Pharm.

[28] Chattopadhyay A, Mondal T, Saha TK, Dey I, Sahu BK, Bhattacharya J (2013). An audit of prescribing practices in CGHS dispensaries of Kolkata, India. IOSR J Dent Med Sci.

[29] Hogerzeil H, Ross-Degnan D, Laing R, Ofori-Adjei D, Santoso B, Chowdhury AA (1993). Field tests for rational drug use in twelve developing countries. Lancet.

[30] Henry CN, Ogaji IJ, Sariem CN (2013). Drug use pattern with standard indicators in Jos University Teaching Hospital Nigeria. West Afr J Pharm.

[31] Kafle K (1992). INRUD drug use indicators in Nepal: Practice patterns in health posts in four districts. INRUD News.

[32] Angamo MT, Wabe NT, Raju N (2011). Assessment of patterns of drug use by using World Health Organization’s prescribing, patient care and health facility indicators in selected health facilities in Southwest Ethiopia. J App Pharm Sci.

[33] Nsimba SED (2006). Assessing prescribing and patient care indicator for children under five years old with malaria and other disease conditions in public primary health care facilities. Southeast Asian J Trop Med Public Health.

[34] Gelders S, World Health Organization. Drug use indicator survey, 1991-Malawi Essential Drugs Programme.1992. http://apps.who.int/medicinedocs/en/d/Js21679en/. Accessed October 18 2015

[35] El Mahalli A, Akl O, Al-Dawood S, Al-Nehab A, Al-Kubaish H, Al-Saeed S (2012). WHO/INRUD patient care and facility-specific drug use indicators at primary health care centres in Eastern province, Saudi Arabia. East Mediterr Health J.

[36] Bimo D (1992). Report on Nigerian field test. INRUD news.

[37] Massele AY, Nsimba SE, Rimoy G (2001). Prescribing habits in church-owned primary health care facilities in Dar Es Salaam and other Tanzanian coast regions. East Afr Med J.

[38] Tomson G (1992). Pilot study of drug use indicators in Sweden. Inrud News.

[39] Awad A, Al-Saffar N (2010). Evaluation of drug use practices at primary healthcare centers of Kuwait. Eur J Clin Pharmacol.

[40] Naseeb TA, Nasser MA (2005). Drug prescribing indicators in primary health care centers in Bahrain. Saudi Med J.

[41] Sharif S, Al-Shaqra M, Hajjar H, Shamout A, Wess L (2008). Patterns of drug prescribing in a hospital in dubai, United arab emirates. Libyan J Med.

[42] Lopes A, Teixeira A, Gurgel M, Miranda M, Oliveira M, Oliveira M (1996). Drug use of evaluation in health services in Fortaleza, Brasil. INRUD News.

[43] Folkedal SF G, Banqueiro EG (1994). Rational Drug Use Field Tests: Experience from Mozambique. INRUD News.

